# Clinicopathological Characteristics of Cancer-Associated Venous Thromboembolism (CAT-VTE) from a Medicolegal Autopsy

**DOI:** 10.3400/avd.oa.22-00034

**Published:** 2022-06-25

**Authors:** Ayako Ro, Norimasa Kageyama, Toshiji Mukai

**Affiliations:** 1 Department of Legal Medicine, St. Marianna University School of Medicine, Kawasaki, Kanagawa, Japan; 2 Tokyo Medical Examiner’s Office, Tokyo Metropolitan Government, Tokyo, Japan

**Keywords:** cancer-associated thrombosis, venous thromboembolism, pulmonary thromboembolism, deep vein thrombosis, medicolegal autopsy

## Abstract

**Objective:** This study aimed to determine the clinicopathological characteristics of cancer-associated venous thromboembolism (CAT-VTE).

**Methods:** A total of 47 cases of lethal pulmonary thromboembolism (PTE) with active cancer were investigated by autopsy records.

**Results:** We studied 22 men and 25 women who were deceased at a mean age of 66±11 years. Nine (19%) patients had recently undergone cancer resection, 14 (30%) were undergoing clinical treatment for cancer, and 24 (51%) were autopsy-proven CAT-VTE. The colon (eight cases), lungs (seven cases), and ovaries (six cases) were frequent sites of a tumor. There were 29 (62%) cases of acute PTE and 18 (38%) of recurrent PTE. The embolic source was detected in 36/39 (92%) cases. Among them, 33 cases were leg deep vein thrombosis (DVT) and 31 were calf-type DVT. Three cases were isolated vena cava thrombi that were present near the tumor. Twenty-three (64%) cases were recurrent DVT.

**Conclusion:** Most of the lethal CAT-VTE cases were induced by the same mechanism as non-CAT-VTE that originated from calf-type DVT with proximal propagation. However, the finding that patients had tumor-related vena cava thrombi suggested that prevention of CAT-VTE requires individualized treatment of patients according to their pathological condition. (This is secondary publication from Jpn J Phlebol 2020; 31(3): 123–129.)

## Introduction

Thrombosis that develops as a cancer complication is referred to as cancer-associated thrombosis (CAT). It is classified under several subtypes, namely, arterial thromboembolism, disseminated intravascular coagulation, non-bacterial thrombotic endocarditis, and venous thromboembolism (VTE).^1)^ CAT is a frequent cause of death in patients with cancer. Among CAT, thromboembolism is the second most common cause of death in patients undergoing outpatient chemotherapy, second only to death from cancer.^2)^ In CAT, VTE associated with acute massive pulmonary thromboembolism (PTE) has a risk of sudden death. We have previously studied medicolegal autopsy cases of sudden death with acute massive PTE.^3–6)^ In this study, we focused on lethal CAT-PTE cases and investigated their clinicopathological characteristics.

## Patients and Methods

We selected 47 patients diagnosed with lethal PTE and active cancer by autopsy, which was performed at the Tokyo Medical Examiner’s Office between 2005 and 2019. During the same period as the present study, 36,123 medicolegal autopsies were performed, and 798 resulted in a diagnosis of PTE. Patients with tumor embolism, nonfatal submassive PTE with cancer, and PTE cases with a previous history of nonactive cancer were excluded from this study. The following clinicopathological characteristics were investigated retrospectively from the autopsy records: age, sex, body mass index (BMI), heart weight, property of cardiac blood, primary site of the tumor, antemortem cancer/VTE treatment, situation of PTE onset, and pathological findings of PTE and deep vein thrombosis (DVT).

The BMI was calculated by dividing the patient’s body weight in kilograms by their height in meters squared, and a BMI <18.5 kg/m^2^ was classified as underweight, 18.5–25 kg/m^2^ as the normal range, and ≥25 kg/m^2^ as obese according to the criteria of the Japan Society for the Study of Obesity.^7)^ The primary site of cancer was evaluated using the Khorana VTE risk score (KRS), which is divided into very high risk (stomach and pancreas), high risk (lungs, lymphoma, gynecological organs, bladder, and testes), and others.^8)^

**Figure 1:**
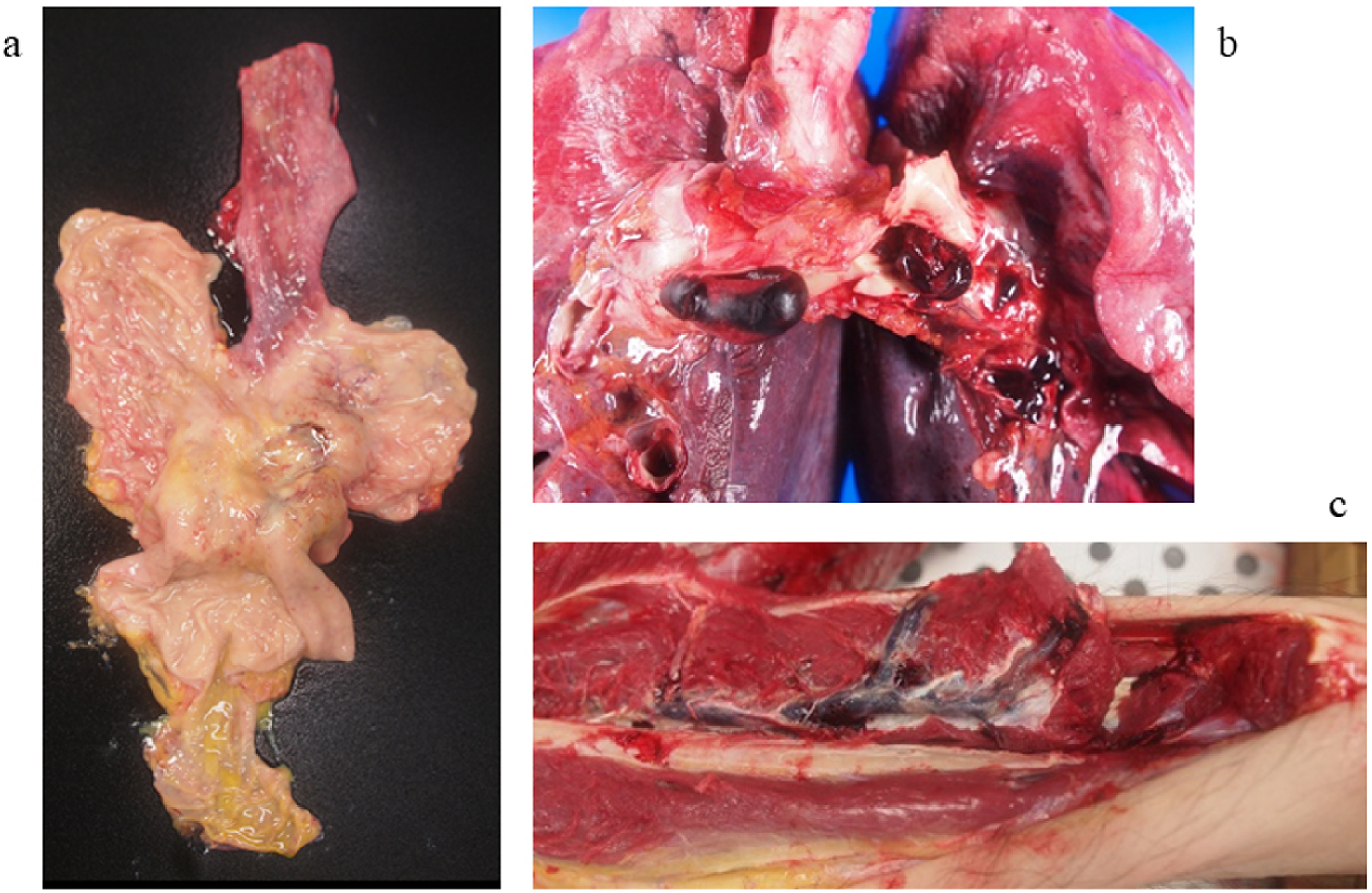
Fig. 1 Representative case of acute type cancer-associated venous thromboembolism due to undiagnosed gastric cancer. (**a**): Large gastric cancer. （**b**）: Massive fresh pulmonary thromboembolism occluding bilateral pulmonary arteries. (**c**): Left fresh calf deep vein thrombosis.

**Figure 2:**
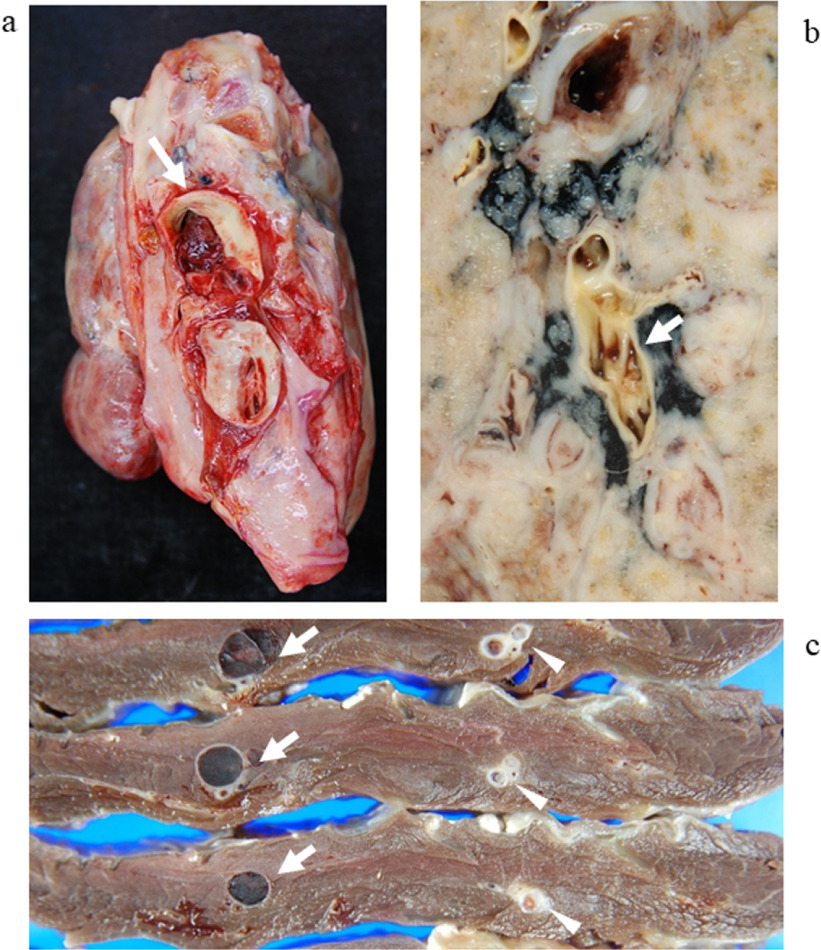
Fig. 2 Representative case of recurrent type cancer-associated venous thromboembolism due to undiagnosed lung cancer. (**a**): The right lung was retracted by massive tumor invasion with proximal pulmonary thromboembolism (arrow). Tumor emboli were not detected. （**b**）: A small artery shows a band-like appearance by organized thrombi (arrow). (**c**): The soleal vein has fresh (arrows) and organized thrombi (arrowheads).

Pathological findings of VTE were confirmed by the PTE location and chronology (disease duration) and the location and classification of the venous thrombosis as the embolic source. Regarding the classification of PTE and venous thrombus, cases with only fresh thrombus were classified as the acute type (**Fig. 1**), cases with a mixture of fresh and organized thrombus were classified as the recurrent type (**Fig. 2**), and cases with only organized thrombus were classified as the previous type. The DVT of the lower extremities was classified as the calf type when the thrombus was confined to the calf deep vein and developed continuously to the proximal veins. DVT was classified as the proximal type when the thrombus was restricted to the proximal deep veins without calf DVT. We further divided patients into the following groups: (1) those who were undergoing cancer treatment and underwent cancer resection within 3 months of death (with recent surgery; surgery group); (2) those who were undergoing cancer treatment and did not undergo surgery within 3 months of death (without recent surgery; treated group); and (3) those who died and had CAT-VTE at autopsy without an antemortem diagnosis of either cancer or VTE (autopsy-proven CAT-PTE; untreated group). The pathological findings of VTE in each group were examined. This study was approved by the ethical committee of the Tokyo Medical Examiner’s Office (2016-5) and St. Marianna University School of Medicine (3489).

## Results

### 1. Patients’ characteristics

This study included 22 men and 25 women with a mean (SD) age of 66.2±10.9 years (38–84 years) (**Table 1**). The mean BMI was 20.6±4.3 kg/m^2^. Among them, 7 (15%) were obese and 14 (30%) were underweight. The mean (SD) cardiac weight was 370±89 g. The nature of cardiac blood was fluid blood only in 32 (68%) patients and a mixture of fluid blood and postmortem clots in 15 (32%) patients.

**Table 1:** Table 1 Clinico-pathological characters of cancer associated pulmonary thromboembolism

Sex	
male	22 (47%)
female	25 (53%)
Age (mean±S.D., years)	66.2±10.9
<60 years (%)	12 (26%)
60≦ ≧80 years (%)	30 (64%)
>80 years (%)	5 (11%)
BMI (mean±S.D., kg/m^2^)	20.6±4.3
<18 (%)	14 (30%)
18.5≦ ≧25 (%)	26 (55%)
>25 (%)	7 (15%)
Site of cancer (%)	
colon	8 (16.7%)
lung	7 (14.6%)
ovary	6 (12.5%)
other	26 (54.2%)
unknown	1 (2.1%)
With cancer metastasis	28 (60%)

### 2. Sites and types of cancer

The primary sites of cancer were as follows: the colon (n=8); lungs (n=7); ovaries (n=6); stomach (n=5); malignant lymphoma (n=4); rectum (n=4); uterus (n=4); kidney (n=2); and the brain, esophagus, pharynx, prostate, intrahepatic cholangiocarcinoma, and pancreas (n=1 each). The origin of the tumor could not be detected in one case because of massive systemic metastasis. According to the KRS, 6 (13%) cases were classified as very high risk and 21 (45%) as high risk. The remaining 20 (42%) cases were not classified using this classification. Twenty-eight （60％） cases had distant metastasis (**Table 1**).

### 3. Medical treatment status of cancer and VTE

Twenty-three (49%) patients were undergoing treatment for cancer, among whom 4 (17%) were hospitalized and 19 (83%) were outpatients (**Table 2**). Of the patients undergoing cancer treatment, 9 were in the surgery group, and the time between surgery and death was within 10 days in 3 patients, within 1 month in 3 patients, and within 3 months in 3 patients.

**Table 2:** Table 2 Relationship between the patient’s VTE pathology and their premortem medical conditions

	Total (n=47)	Under premortem cancer treatment		Autopsy-proven CAT-PTE
		with recent surgery (n=9)	without recent surgery (n=14)	(n=24)
Place of onset				
inhospital	4 (9%)	3 (33%)	1 (7%)	0
outpatients	43 (91%)	6 (66%)	13 (93%)	24 (100%)
Pulmonary thrombi pathology				
acute	29 (62%)	8 (89%)	7 (50%)	14 (58%)
recurrent	18 (38%)	1 (11%)	7 (50%)	10 (42%)
Embolic source pathology (n=36)				
acute	12 (33%)	3 (43%)	3 (27%)	6 (33%)
recurrent or previous	24 (67%)	4 (57%)	8 (73%)	12 (67%)
Location of embolic source (n=36)				
leg deep vein	33 (92%)	7 (100%)	10 (91%)	16 (89%)
IVC	2 (6%)	0	1 (9%)	1 (6%)
SVC	1 (3%)	0	0	1 (6%)
Detailed characters of leg DVT (n=33)				
Leg DVT laterality				
bilateral	28 (85%)	5 (71%)	9 (90%)	14 (88%)
unilateral	5 (15%)	2 (29%)	1 (10%)	2 (13%)
Leg DVT location				
calf type	31 (94%)	7 (100%)	10 (100%)	14 (88%)
proxymal only	2 (6%)	0	0	2 (13%)

All patients in the surgery group who developed PTE within 10 days after surgery were hospitalized. All 6 patients in the surgery group who developed PTE after 10 days postoperatively were discharged from the hospital and developed PTE at home.

There were 14 patients in the treatment group, all of whom were outpatients, except for one. Six patients were treated with chemotherapy. One of the patients undergoing cancer treatment had been diagnosed with DVT and PTE before death and was treated with an inferior vena cava filter and oral anticoagulants, but died suddenly of recurrent PTE. No other patients undergoing treatment for cancer had a prenatal diagnosis of VTE.

Twenty-four (51%) patients were in the untreated group with autopsy-proven CAT-VTE.

### 4. Pathological characteristics of VTE

All cases of CAT-VTE were massive PTE, and fresh thrombus was located in at least one lobar artery. PTE was classified as the acute type in 29 (62%) cases and the recurrent type in 18 (38%) cases (**Table 2**).

The embolic source was investigated in 39 cases of CAT-VTE, and venous thrombi were detected in 36 (92%) cases. Venous thrombi were classified as the acute type in 12 (33%) cases, the recurrent type in 23 (64%) cases, and the previous type in 1 (3%) case. The leg was the most frequent site of the embolic source in 33 (92%) cases. Among them, 28 (85%) were bilateral and 5 (15%) were unilateral. Thirty-one (94%) cases of leg DVT were the calf type. Among cases with a detailed description of the location of thrombi in the leg deep veins, the soleal vein was the most frequent thrombi site.

An embolic source other than in a leg deep vein was found in 3 (9%) patients. These patients each had a superior vena cava thrombus associated with lung cancer, inferior vena cava thrombus with renal cancer, and inferior vena cava thrombus associated with peritoneal malignant lymphoma. No patients showed direct invasion of the tumor into the veins. Two patients had venous thrombi in leg deep veins and non-deep veins, with one thrombus at a dural venous sinus and the other thrombus at a superficial leg vein.

### 5. Medical treatment status and VTE pathology

Statistical comparison of the groups was not performed in this study because of the small number of patients. However, the embolic source in the surgery group was only the calf type. Additionally, the percentage of the acute type in PTE and DVT was higher in the surgery group than in the treated and untreated groups (**Table 2**).

## Discussion

Several factors cause thrombophilia in patients with cancer. Namely, in addition to the state of coagulopathy, inflammation, and hypoxia caused by cancer tissue, various substances are produced by induction of cancer proliferation genes, causing thrombi formation.^1)^ For this reason, cancer is one of the VTE risk factors. Thus, a Japanese cohort study of VTE showed that 27% of patients with VTE had a history of cancer and that a higher mortality rate was observed in patients with cancer than in those without it.^9)^ There has been only one epidemiological report of the CAT-PTE in Japanese autopsy cases by Sakuma et al., to the best of our knowledge, who showed that the incidence of PTE in patients with cancer was 2.3%.^10)^ However, this report was based on pathological autopsies, and 71% of the cases were non-critical submissive PTE, which was detected as an accessory finding of death from cancer.^10)^ The pathological condition of CAT-VTE changes from isolated calf DVT, followed by asymptomatic submassive PTE, and finally to lethal acute massive PTE. In the present study, we reviewed medicolegal autopsy cases to investigate the pathophysiology of acute massive PTE of CAT-VTE, which resulted in sudden death. To the best of our knowledge, this is the first report of CAT-VTE in lethal acute massive PTE in Japan.

The incidence of CAT in patients with PTE detected by medicolegal autopsy was 5.8% in this study, which is much lower than that in the above-mentioned Japanese cohort study.^9)^ In our previous report of an autopsy, the CAT-PTE accounted for only 3.1% of all PTE cases.^3)^ This finding is mainly due to the nature of pathological and medicolegal autopsies. Namely, in medicolegal autopsies that deal with unexpected deaths, patients with advanced cancer who usually have been diagnosed and treated in hospitals before death are rarely included. Indeed, cancer death accounts for only 5.2% of all medicolegal autopsies^11)^ compared to 66% of all pathological autopsies.^10)^ However, the actual incidence of CAT-PTE in medicolegal autopsies could be slightly higher than that found in the present report. The reason for this difference in incidence could be because the CAT-PTE cases in which the autopsy record described only “cancer death” as the cause of death were not included in this study.

Epidemiological characteristics of CAT-VTE in our study are similar to those of the Japanese VTE cohort,^9)^ with a mean age of approximately 60 years and a slightly higher number of women. The KRS is commonly used to estimate risk factors of CAT-VTE.^8)^ Among the KRS risk factors, we only investigated the site of cancer and BMI because other factors of the score are blood biochemistry data, which are not reliable in autopsy samples. The original KRS score indicates that a BMI >35 kg/m^2^ is a CAT-VTE risk factor.^8)^ In this study, we used a BMI >25 kg/m^2^ as a criterion for obesity according to a previous Japanese study.^12)^ Hiraide et al. reported that 28% of patients with lung CAT-VTE were obese, and this was considered a risk factor for CAT-VTE.^12)^ However, in the present study, only 15% of the patients were obese, and 30% were underweight. Obesity is a VTE risk factor with or without cancer. Our previous study of massive PTE in medicolegal autopsy without active cancer showed that the proportion of obesity was 30%.^3)^ Our present and previous results suggest that patients with CAT-PTE tend not to be obese, but underweight, as reflected by their cancer cachexia.

Regarding the site of cancer, 58% of the cases were classified as high- or very high-risk groups according to the KRS. The incidence of CAT-VTE in pathological autopsies is high in hematogenous tissue, the lungs, ovaries, pancreas, and biliary system, and low in the liver.^10)^ In clinical cases, the most common site of cancer is the lungs, followed by the colon, hematopoietic organs, the uterus, and the ovaries.^13)^ The results of the present study are consistent with these reports.

Elevated levels of blood coagulation factors are usually observed in patients with cancer.^14)^ Among them, D-dimer is a clinically important marker for the diagnosis of VTE. We could not perform blood coagulation tests because these tests are not as reliable in autopsy samples as biochemical tests. Therefore, we studied the property of cardiac blood to evaluate coagulability at autopsy. Human blood usually clots after death. However, blood is fluid in sudden-death cases such as PTE. A suggested mechanism of this phenomenon is that excessive agonal release of tissue plasminogen activator from vascular endothelium dissolves the postmortem coagulated blood in sudden death.^15)^ Speaking from experience, most PTE cases without cancer show fluid cardiac blood at autopsy. However, the current study showed that 32% of patients with CAT-PTE had clotted blood. These results may reflect the hypercoagulability characteristic of CAT-PTE.

From a clinical viewpoint, approximately half of the patients had autopsy-proven CAT-VTE in which cancer and VTE were first discovered at autopsy. Additionally, medical institutions had no opportunity to be involved in treatment for CAT-VTE before death. However, the other half of the patients, except for one, were treated for cancer but had not been diagnosed with VTE before death. Among them, 17% of patients with CAT-PTE had undergone recent cancer resection in this study. Moreover, all the patients with CAT-PTE within 10 days from surgery were still in hospital. Because hospitalization and recent surgery are VTE risk factors, prophylaxis is required for patients who had recent surgery, regardless of whether they have cancer.^16)^ Detailed medical information on whether these patients received VTE prophylaxis in the hospital was unavailable because antemortem medical care and postmortem autopsy examinations were performed in different facilities. However, the finding of several hospital deaths perioperatively in patients with cancer in this study indicates the importance of perioperative VTE prophylaxis in patients with cancer. The only embolic source in the surgery group was the calf type, and the percentage of the acute type in PTE and DVT was higher in the surgery group than in the treated and untreated groups. In our previous study, we compared inpatients with and without cancer with outpatients with and without medical treatment.^4)^ We found that 66% of inpatients had the acute type, whereas 82% of outpatients had the recurrent type.^4)^ These findings suggest that, in postoperative patients, not only cancer-induced hypercoagulability, but also venous stasis in the lower extremities due to perioperative bed rest, may have strongly affected thrombi formation.

A total of 83% of patients with CAT-PTE undergoing cancer treatment were outpatients in the present study. Sakamoto et al. reported that 66% of CAT-VTE occurred out of hospital, and outpatients had a higher rate of recurrent VTE than inpatients.^13)^ Additionally, patients with active cancer have a higher risk of VTE recurrence, bleeding, and death than patients without active cancer.^13)^ Therefore, prophylaxis for VTE is necessary for outpatients and inpatients with cancer. However, one patient in our study who was diagnosed with VTE and PTE died due to developing PTE, despite continuous prophylaxis for VTE with medication. This finding suggests the difficulty of providing appropriate outpatient coagulation control in patients with cancer. Therefore, selecting appropriate individual prophylaxis for a large number of cancer outpatients with cancer remains difficult.

Regarding the embolic source of CAT-PTE in our study, 92% of patients had lower extremity DVT, 85% had bilateral DVT, and 94% had the lower leg type of DVT. Based on our previous studies of fatal PTE autopsies, we propose a mechanism in which DVT primarily occurs in calf veins, especially at the soleus vein, because of venous stasis in the lower extremities, and develops proximally to form a large thromboembolus at the proximal vein.^4–6)^ The present study indicated that, even in patients with CAT-PTE, most VTE cases have the same mechanism as those without cancer. Yokoi et al. reported that the most frequent site of CAT-DVT was below the knee (up to 45%) and that there was no significant difference in the rate of concomitant PTE between patients with DVT with or without malignancy.^17)^ Therefore, the calf vein is the primary site of DVT in patients with CAT-VTE, similar to patients with VTE without cancer. In the present study, 3 patients had an isolated vena cava thrombus without leg DVT. All thrombi in these patients were located near tumors. Furthermore, in these patients, the mechanism of thrombi formation was considered to be based on venous compression by the tumor or focal changes in blood coagulability by tumor cells, and not on venous stagnation in the leg. Although the number of cases was small, we consider that a venous thrombus near the site of cancer as an embolic source could be a characteristic of CAT-VTE. We also found that 2 patients had thrombi in the superficial vein or dural sinus, which is not usually observed in PTE without cancer. These results may reflect the systemic hypercoagulability caused by CAT-VTE, similar to the presence of postmortem clots in the heart.

CAT involves multiple mechanisms that are different from thrombosis without cancer.^1)^ The present study suggests that in case of CAT-VTE prophylaxis, we should consider not only venous stagnation, but also multiple factors, such as the physiological nature of cancer—the cancer site and size. The patient’s treatment status should also be considered, such as whether the patient is hospitalized and whether the patient is undergoing surgery or chemotherapy. Therefore, the CAT-VTE prevention should be achieved according to the patient’s individual condition.

This study has several limitations. Because this was a retrospective study of autopsy reports, detailed information, such as the histological cancer type and detailed location of leg DVT, could not be obtained. Additionally, because of the difference in facilities between medical care and medicolegal autopsies, detailed information on cancer care (e.g., diagnostic methods, biochemical data, surgical procedures, therapeutic agents, and VTE prevention methods) could not be obtained as well. Therefore, we could not sufficiently compare the results of autopsies with those of medical treatment, which is important for clinical application of this study’s results. In addition to these limitations, this report provides useful information about the clinicopathological characteristics of rarely known lethal CAT-VTE.

## Conclusion

This study investigated the clinicopathological characteristics of CAT-VTE in medicolegal autopsy. We found that 90% of the embolic sources were DVT of the lower extremities, and the majority was of the calf type. Therefore, the prevention of venous stasis of the leg is as equally important in the prevention of VTE with and without cancer. Finally, the CAT-VTE prevention requires individualized treatment of patients according to their pathological condition.

## Notes

This is secondary publication from Jpn J Phlebol 2020; 31(3): 123–129.

## References

[1] Mukai M, Oka T. Mechanism and management of cancer-associated thrombosis. J Cardiol 2018; 72: 89-93. 29588087 10.1016/j.jjcc.2018.02.011

[2] Khorana AA, Francis CW, Culakova E, et al. Thromboembolism is a leading cause of death in cancer patients receiving outpatient chemotherapy. J Thromb Haemost 2007; 5: 632-4. 17319909 10.1111/j.1538-7836.2007.02374.x

[3] Ro A, Kageyama N, Tanifuji T, et al. Autopsy-proven untreated previous pulmonary thromboembolism: frequency and distribution in the pulmonary artery and correlation with patients’ clinical characteristics. J Thromb Haemost 2011; 9: 922-7. 21294826 10.1111/j.1538-7836.2011.04225.x

[4] Ro A, Kageyama N, Tanifuji T, et al. Pulmonary thromboembolism: overview and update from medicolegal aspects. Leg Med (Tokyo) 2008; 10: 57-71. 18037329 10.1016/j.legalmed.2007.09.003

[5] Ro A, Kageyama N. Clinical significance of the soleal vein and related drainage veins, in calf vein thrombosis in autopsy cases with massive pulmonary thromboembolism. Ann Vasc Dis 2016; 9: 15-21. 27087868 10.3400/avd.oa.15-00088 PMC4807210

[6] Ro A, Kageyama N, Mukai T. Pathophysiology of venous thromboembolism with respect to the anatomical features of the deep veins of lower limbs: a review. Ann Vasc Dis 2017; 10: 99-106. 29034034 10.3400/avd.ra.17-00035 PMC5579784

[7] Japan Society for the Study of Obesity. Guidelines for the management of obesity disease 2016. http://www.jasso.or.jp/data/magazine/pdf/chart_A.pdf (accessed 2021-6-25). (in Japanese)

[8] Khorana AA, Kuderer NM, Culakova E, et al. Development and validation of a predictive model for chemotherapy-associated thrombosis. Blood 2008; 111: 4902-7. 18216292 10.1182/blood-2007-10-116327 PMC2384124

[9] Nakamura M, Miyata T, Ozeki Y, et al. Current venous thromboembolism management and outcomes in Japan. Circ J 2014; 78: 708-17. 24401573 10.1253/circj.cj-13-0886

[10] Sakuma M, Fukui S, Nakamura M, et al. Cancer and pulmonary embolism: thrombotic embolism, tumor embolism, and tumor invasion into a large vein. Circ J 2006; 70: 744-9. 16723797 10.1253/circj.70.744

[11] Tokyo Medical Examiner’s Office. In: Tokyo Medical Examiner’s Office. Business summary of Tokyo Medical Examiner’s Office in 2018. Tokyo, 2018: 30. (in Japanese)

[12] Hiraide M, Shiga T, Minowa Y, et al. Identification of risk factors for venous thromboembolism and evaluation of Khorana venous thromboembolism risk assessment in Japanese lung cancer patients. J Cardiol 2020; 75: 110-4. 31416782 10.1016/j.jjcc.2019.06.013

[13] Sakamoto J, Yamashita Y, Morimoto T, et al. Cancer-associated venous thromboembolism in the real world: from the COMMAND VTE Registry. Circ J 2019; 83: 2271-81. 31548438 10.1253/circj.CJ-19-0515

[14] Yamashita Y, Wada H, Nomura H, et al. Elevated fibrin-related markers in patients with malignant diseases frequently associated with disseminated intravascular coagulation and venous thromboembolism. Intern Med 2014; 53: 413-9. 24583428 10.2169/internalmedicine.53.1102

[15] Ro A. Blood coagulation in autopsy cases: thrombus and postmortem clotting. Thrombosis Medicine 2019; 9: 12-6. (in Japanese)

[16] Ito M, Ikeda M, Ishibashi H, et al. Guidelines for Diagnosis, Treatment and Prevention of Pulmonary Thromboembolism and Deep Vein Thrombosis (JCS 2017), 2017: 8. (in Japanese) https://js-phlebology.jp/wp/wp-content/uploads/2019/03/JCS2017_ito_h.pdf (accessed 2021-6-25)

[17] Yokoi K, Hara M, Ueda Y, et al. Epidemiological and outcome data in Japanese patients with deep vein thrombosis with and without malignancy. Heart Vessels 2017; 32: 1469-77. 28741216 10.1007/s00380-017-1025-0

